# Helmuth’s System of Surgery

**Published:** 1887-01

**Authors:** 


HELMUTH'S SYSTEM OF SURGERY.

The fifth edition of this well-known work is promised by the Hahnemann Publishing House in November.

